# PROFILES OF EXECUTIVE FUNCTIONING FOLLOWING TRAUMATIC BRAIN INJURY AND STROKE USING THE ASSESSMENT OF PARTICIPATION AND EXECUTIVE FUNCTIONS: COMBINED CROSS-SECTIONAL AND LONGITUDINAL DESIGNS

**DOI:** 10.2340/jrm.v56.12427

**Published:** 2024-01-18

**Authors:** Rotem ELIAV, Sivan HASSON, Rachel KIZONY

**Affiliations:** 1 Department of Occupational Therapy, Faculty of Welfare and Health Sciences, University of Haifa, Haifa; 2 Department of Occupational Therapy, Loewenstein Rehabilitation Medical Center, Ra’anana; 3 Department of Occupational Therapy, Sheba Medical Center, Tel-Hashomer, Israel

**Keywords:** executive function, inpatient, rehabilitation, stroke, traumatic brain injury

## Abstract

**Objectives:**

The Assessment of Participation and Executive Functions (A-PEX) evaluates executive functioning through daily participation in complex daily activities. This study examines its ability to discriminate between executive functioning profiles post-traumatic brain injury and post-stroke and its sensitivity to changes.

**Design:**

Cross-sectional with a longitudinal component.

**Patients:**

Adults with post-traumatic brain injury (*n* = 28) and post-stroke (*n* = 26) in a rehabilitation facility.

**Methods:**

Patients were administered the A-PEX, Multiple Errands Test-Hospital version and Color Trail Test at 2 time-points 1 month apart. The Montreal Cognitive Assessment was administered at the first time-point, and Executive Functions Performance Test’s Internet-based Bill Payment subtest at the second. The analysis used Mann–Whitney and Wilcoxon signed-rank tests.

**Results:**

The stroke group’s A-PEX scores were higher than the traumatic brain injury group’s at the first time-point (*p* < 0.05). No differences were found in the other assessments. Within-group differences in both groups were significant in the A-PEX (–3.7 < *r* < – 2.3, *p* < 0.05) and Multiple Errands Test-Hospital version (–3.4 < *r* < –3.3, *p* < 0.01).

**Conclusion:**

The A-PEX may provide valuable information about the uniqueness of executive functioning profiles and patients’ progress.

LAY ABSTRACT

Traumatic brain injury and stroke are health conditions that impair executive functions. Executive functions are higher-level cognitive abilities, such as problem-solving and planning, which are needed for participating in daily activities. The 2 health conditions differ in how the resulting executive functioning deficits affect daily participation, so it is important to understand their commonalities and differences. The Assessment of Participation and Executive Functions (A-PEX) is a tool that evaluates executive functioning and how they influence daily participation in activities such as shopping. Using the A-PEX to better characterize the executive functioning profiles in both health conditions, this study found that post-stroke patients could function better than post-traumatic brain injury patients, despite being older. Traditional assessment tools had not found such differences. The results indicate that the A-PEX can provide important information about executive functioning profiles post-traumatic brain injury compared with post-stroke.

Executive function (EF) deficits are common sequelae of acquired brain injury (ABI), which includes traumatic brain injury (TBI) and stroke (1), and often lead to long-term participation restrictions in complex daily activities, such as Instrumental Activities of Daily Living (IADL), leisure and social activities (2–6). The prevalences of EF deficits following TBI and stroke during the subacute recovery phase are 48% and 40%, respectively (2, 7, 8), and persist years after onset (9, 10). Executive functions can be “cold”, indicating cognitive domains (e.g. planning or monitoring), or “hot”, indicating behavioural domains (e.g. social judgement or emotional regulation) (2, 11).

Following stroke, EF domain deficits include impaired initiation and information generation, and behavioural domain deficits include hypoactivity with disinterest and anticipation loss (8). In TBI, deficits include impaired inhibition, monitoring, planning, organisation, cognitive flexibility and working memory in the cognitive domain (11–13) and irritability, disinhibition, apathy and emotional lability in the behavioural domain (14). There may be overlaps in EF deficits among patients post-TBI and those post-stroke, such as apathy, which may be less common post-stroke but still exists (2). Clinical practice guidelines differ on whether TBI and stroke should be addressed separately (15–17). However, considering this variability, assessment tools must be sensitive to various executive functioning profiles.

Performance-based assessments (18, 19) aim to increase the ecological validity (e.g. resemblance to daily life) (20) of EF assessments. They simulate real-world activities through structured tasks rather than everyday open-ended activities, raising concerns about their ability to accurately examine executive functioning deficit effects on actual participation (20–22) or distinguish between executive functioning profiles. Using actual everyday participation to evaluate EFs ensures that the tasks are relevant to the patient’s daily function, making the assessment more ecologically valid and perhaps sensitive to different profiles (21, 23).

Following ABI, patients typically spend long periods in inpatient rehabilitation facilities (24), participating in open-ended activities, such as maintaining relationships with fellow patients or scheduling an appointment with the physician. These activities are similar to home- and community-based activities, but differ in scale and complexity. Based on the International Classification of Functioning, Disability and Health (ICF) model (25), which emphasizes daily participation in current context, we propose “inpatient participation” to mean instrumental activities of daily living (IADL), leisure activities, and social interactions during hospitalization.

We designed the Assessment of Participation and Executive Functions (A-PEX) to assess executive functioning through inpatient participation. Capturing the manifestation of executive functioning in the patient’s actual daily participation in current context overcomes the limitations mentioned above of existing tools that are using structured tasks. It may yield new insights into the executive functioning profiles of TBI and stroke, possibly leading to more accurate treatment planning, progress measurement and outcome prediction. This study aimed to compare the A-PEX with frequently used executive functioning and EF assessments in: (*i*) its ability to discriminate between profiles of executive functioning as manifested in daily participation of adults undergoing inpatient rehabilitation following TBI and stroke; and (*ii*) sensitivity to changes in executive functioning as manifested in performance of daily activities and participation. We hypothesize that: (*i*) the A-PEX will be able to better discriminate between TBI and stroke executive functioning profiles compared with frequently used EF assessments; (*ii*) changes in executive functioning as manifested in daily participation during inpatient rehabilitation will be captured by the A-PEX.

## METHODS

### Study design

This study used a cross-sectional design with a longitudinal component. The Loewenstein Medical Centre Review Board (#0028-18-LOE) and the University of Haifa Ethics Committee (#161/19) approved the study protocol. All participants provided written consent prior to participation. Data collection was carried out between April 2020 and September 2022.

### Patients

The study sample comprised 54 patients hospitalized in an inpatient rehabilitation medical centre, divided into 2 groups. The TBI group included 28 patients (some were included in a study to validate the A-PEX) (26); the stroke group included 26. Inclusion criteria for both groups were diagnosis of moderate-to-severe TBI or stroke, confirmed by imaging (according to their medical records); ages 18–70 years; able to understand the assessment tool instructions; had preserved basic cognitive abilities that are needed to undergo the assessments administered in the current study, as determined by 3 domains of the Loewenstein Occupational Therapy Cognitive Assessment: orientation (scoring 6/8 or above), visual perception (3/4 or above), and spatial perception (2/4 or above) (27); had at least 1 functional upper extremity, as determined by their occupational therapist; and had intact or corrected vision. Exclusion criteria were: a history of epilepsy, drug use or psychiatric or other neurological disorders affecting cognitive function. Sample size was calculated using G*Power software (Dusseldorf, Germany) for between-groups comparisons; 1 tail, with medium-large effect size (d = 0.65), α = 0.05 and power = 0.80. The sample size needed was *n* = 30 in each group. Due to the SARS-CoV-2 (COVID-19) pandemic the sample size was smaller. Therefore, we report on effect sizes and acknowledge the sample size as a limitation.

### Procedure

All patients who were eligible according to the inclusion criteria were approached. Seven patients with TBI and 3 patients with stroke declined to participate in the study. Once patients were eligible and agreed to participate in the study, we administered the A-PEX, Multiple Errands Test-Hospital Version (MET-HV) and Color Trail Test (CTT) at 2 time-points, 1 month apart. The Montreal Cognitive Assessment (MoCA) was administered at the first time-point, and the Internet-based version of the Executive Functions Performance Test (EFPT) Bill Payment subtest at the second time-point. Administration of the assessments took place in the rehabilitation facility grounds or in a quiet room, depending on the assessment requirements. The assessors, who were not blind to the type of injury, were experienced occupational therapists that underwent 5 1-h training sessions. The assessors were able to consult the first author when needed. Demographic information, medical data, and functional measure scores for the Functional Independence Measure (FIM) were obtained from the patients’ medical records. During data collection all patients underwent the usual rehabilitation programme that included 5 days a week of occupational and physical therapy, and psychological and medical treatment as needed. Furthermore, participants in both groups had the same conditions in terms of inpatient participation, i.e. they had the same opportunities for social interaction with people in similar age groups, for participation in leisure activities, etc.

### Measures

*Assessment of participation and executive functions.* The A-PEX was originally developed for adults following TBI and then expanded to stroke patients. It evaluates executive functioning as reflected in inpatient participation, mainly leisure, social and IADL, for adults following ABI hospitalized in a specialist inpatient rehabilitation facility (26). The clinician scores the A-PEX based on semi-structured interviews with the patient or primary caregiver and clinical observations over up to 2 weeks of the patient’s inpatient participation (i.e. performance of open-ended meaningful activities in current context). The A-PEX includes 6 functional domains, each of which includes several complex daily activities: (1) treatment-schedule management, (2) medical and social-status management, (3) financial management, (4) mobile phone use, (5) social interaction and (6) leisure. Each domain comprises several items. All activities in each functional domain require EFs and their integration. Fifty-one items relevant during the initial stages of inpatient rehabilitation were analysed for this study. Each item is scored for performance consistency and efficiency on a scale from 1 (does not perform/ineffective performance) to 3 (performs consistently/effective performance). Consistency and efficacy scores are multiplied for each item, then a mean score of all items per functional domain is calculated. Final scores for each functional domain range from 1 to 9. Higher scores indicate better performance.

The A-PEX also includes 2 general EF scales, hot and cold, that are required for the performance of the complex daily activities assessed in the A-PEX’s functional domains. Each EF scale is scored separately based on the observation on activities included in the functional domains: the EF cognitive scale (e.g. self-monitoring, problem-solving, cognitive flexibility; cold EF) and the EF behavioural scale (e.g. emotional lability, perseveration; hot EF). Each component is rated on a scale between 1 (always) and 5 (never), based on its effect on inpatient participation. A mean score is then calculated for each EF scale, rendering a final score between 1 (severe EF deficits) and 5 (no EF deficits).

The A-PEX content validity, internal consistency and initial inter-rater reliability have been established, as has known-group validity between TBI and inpatients with no cognitive deficits (hospitalized due to orthopaedic or spinal cord injury). Convergent validity with the MET-HV and the CTT have been established for post-TBI inpatients in the subacute phase (26).

*Multiple Errands Test-Hospital Version.* The MET-HV is a performance-based assessment to evaluate the effect of EF deficits on everyday functioning in a hospital environment (28). It consists of tasks such as running errands or obtaining information in a hospital setting. Scores range between 0 and 36; lower scores indicate better performance. Inter-rater reliability was good, ranging from 0.81 to 1.00, and internal consistency was satisfactory, at 0.77. Criterion validity was established, and significant differences were found between healthy adults and adults with ABI (28).

*Color Trails Test – a measure of EF.* The CTT is a language-free version of the Trail-Making Test (TMT) and consists of 2 parts. In CTT1, which examines sustained visual attention (29), the participant connects circles in an ascending numbered sequence (from 1 to 25); in CTT2, which examines processing speed, sequence alternation, cognitive flexibility, visual search and executive functioning, the participant connects those numbers while alternating between 2 colours. In this study, CTT2 was used because it is thought to be a more sensitive indicator of executive dysfunction (29, 30). Scores were generated by converting the time required to complete the task to a standard *t*-score adjusted for age and education. Higher scores indicate better performance. The interference index was calculated as a “pure” measure of the interference attributable to EFs required to perform CTT2 using the following formula: [(CTT2–CTT1)/CTT1] (29). The CTT’s concurrent validity with the TMT was excellent (31). Test–retest reliability was excellent for the CTT2 (29).

*Montreal Cognitive Assessment.* The MoCA was designed to detect mild cognitive impairment. It evaluates 8 cognitive domains, such as attention, memory, orientation and EFs. The maximum score is 30, indicating higher cognitive abilities. It has good internal consistency (32). The MoCA has discriminant validity between healthy adults and adults with mild cognitive impairment (cut-off value 26), and it has been used to examine cognitive impairment profiles of TBI and stroke patients (32, 33).

*Executive Functions Performance Test Internet-Based Bill Payment Subtest.* The EFPT is a standardized, performance-based assessment of EF using structured activities commonly used with TBI and stroke populations. It includes 4 tasks: simple cooking, telephone use, medication management, and bill payment (19). This study used an Internet-based version of the Bill Payment subtest (34). This version requires the person to pay 2 bills using a credit card on software that simulates an Internet site. Performance is scored using a scale from 0 to 25, ranging from no cues required (independent = 0 points) to the need to perform the task for the participant (dependent=25 points). The Internet-based version’s criterion and construct validity have been established for adults with TBI and stroke (34, 35).

*Functional Measure for Basic Activities of Daily Living to Describe Sample Characteristics: Functional Independence Measure.* The FIM is commonly used in inpatient rehabilitation facilities to assess basic activities of daily living. It comprises 18 items (13 motor and 5 cognitive). Scores range from 1 to 7 for each item; final (sum of items) scores range from 18 to 126; higher scores indicate more independence (36). Research has found the FIM to be a valid measure of functional independence for TBI and stroke patients (37).

*Screening Measure for Basic Cognitive Abilities: Loewenstein Occupational Therapy Cognitive Assessment.* The Loewenstein Occupational Therapy Cognitive Assessment (LOTCA) was designed to provide a preliminary cognitive profile for patients with ABI (27). It comprises 6 cognitive domains: (1) orientation, (2) visual perception, (3) spatial perception, (4) praxis, (5) visuomotor construction, and (6) thinking operations. Orientation and visual and spatial perception were used to screen for both groups. The LOTCA’s psychometric properties have been established in various populations (26).

### Statistical analysis

Data analysis was conducted using IBM SPSS (Version 25; Armonk, NY: IBM Corp).According to the Shapiro–Wilk test, the data were not normally distributed (38); therefore, we used non-parametric tests for statistical analysis. Participants’ characteristics and descriptive statistics in both groups were summarized using median, interquartile range or percentages.

Between-group differences were examined using Mann–Whitney *U* tests and within-group differences between the first and second time-points using Wilcoxon signed-rank tests. Effect sizes were calculated using Cohen’s *r* = *Z*/√N for non-parametric tests (with *Z* from the Mann–Whitney *U-*test or Wilcoxon signed-rank test) to understand the extent of the between- and within-group differences and interpreted the Cohen’s *r* values as large (> 0.50), medium (0.50–0.30) or small (< 0.30) effects (39).

At the first time-point, all patients in both groups completed the A-PEX, MET-HV, CTT and MoCA except for 1 in the stroke group who declined to undergo the MET-HV. At the second time-point, 3 TBI group patients did not complete all assessments (2 were discharged early, and 1 withdrew from the study). The MET-HV was not administered to 2 additional TBI patients (1 declined to undergo the assessment and 1 due to technical difficulties) or the CTT to 2 others (due to technical difficulties). In the stroke group, 1 patient declined to complete the MET-HV. All analyses for the second time-point included only patients who underwent all the assessments in both time-points. Mann–Whitney tests were used for continuous variables and χ^2^ tests for categorical variables for sensitivity analysis to test for significant differences in study variables between patients who underwent all the assessments at both time-points and those who did not.

## RESULTS

Table I presents demographic and injury-related characteristics for both groups. The TBI group was significantly younger, had lower FIM cognitive scores and was administered the first assessment later post-injury. Motor vehicle accidents were the most common cause of injury in the TBI group, affecting 60.7% of participants. Other injury causes included work-related (14.3%), falls (7.1%), violence (10.7%) and sport (7.1%). In the TBI group, 22 (78.6%) participants had experienced extensive cerebral injury (either diffuse axonal injury or multiple cerebral contusions), and 6 (21.4%) had localized cerebral contusions. In the stroke group, 19 (73.1%) participants had experienced an ischaemic stroke, and 7 (26.9%) had a haemorrhagic stroke. Twelve (46.2%) participants’ strokes affected the right hemisphere, and 12 (46.2%) affected the left hemisphere. Two (7.7%) participants were affected on both sides. There were no significant differences in demographic data in the sensitivity analysis between patients who underwent all assessments at both time-points and those who did not. Due to the age differences between the groups, correlation analysis was performed between age and assessments administered in this study including the A-PEX. Except for the MoCA, which significantly correlated with age in the stroke group (*r =* –0.39, *p =* 0.04), there were no significant correlations.

**Table I:** Participant demographics and characterization

Demographic	TBI group (*N* = 28)	Stroke group (*N* = 26)	*z, p*-value	
Age, year, median (IQR)	33.0 (24.25–49.75)	57.0 (52.75–65.25)	–4.520, > 0.001	
Education, year, median (IQR)	12.0 (12.0–16.0)	12.5 (12.0–15.25)	–1.000, 0.317	
FIM, median (IQR)				
FIM motor (13–91)	61.0 (49.75–71.5)	65.5 (50.0–72.5)	–0.572, 0.568	
FIM cognitive (5–35)	26.0 (23.0–29.0)	30.0 (28.0–33.0)	–3.887, > 0.001	
FIM total (18–126)	84.5 (74.0–97.5)	97.5 (79.5–103.5)	–1.637, 0.102	
Days since injury, median (IQR)	38.0 (29.0–51.5)	28.5 (21.75–31.75)	–2.176, 0.030	
Days since admission, median (IQR)	12.0 (8.0–17.75)	13.0 (10.25–16.75)	–0.642, 0.521	
			χ^2^, *p*-value	Fisher’s exact
Sex, *n* (%)			1.23, 0.267	0.346
Male	23 (82.1)	18 (69.2)		
Marital status, *n* (%)			12.84, > 0.01	> 0.001
Married	13 (46.4)	25 (96.2)		
Mobility, *n* (%)			0.54, 0.908	
Wheelchair	17 (60.7)	16 (61.5)		
Walking aid	6 (21.5)	7 (26.9)		
Walking independently	5 (17.9)	3 (11.5)		

TBI: traumatic brain injury; IQR: interquartile range; FIM: Functional Independence Measure.

Tables II and III present the Mann–Whitney *U-*test results for the A-PEX at the first and second time-points, respectively. At the first time-point, the TBI group scored significantly lower than the stroke group on both EF scales of the A-PEX and all functional domains except mobile phone use. Medium-to-large effect sizes were found in 3 functional domains (medical and social-status management, social interaction, and leisure) and in both EF scales. At the second time-point, the TBI group scored significantly lower than the stroke group in the social interaction functional domain and both EF scales. No significant differences were found between the groups at either time-point in the MET-HV and CTT (Tables IV and V).

**Table II:** Between-group comparisons of the Assessment of Participation and Executive Functions (A-PEX) scores: first time-point

Assessment of Participation and Executive Functions	TBI group (*N* = 28) Median (IQR)	Stroke group (*N* = 26) Median (IQR)	*z*	*p*-value	Cohen’s *r*
Treatment-schedule management	6.4 (2.2–8.6)	8.5 (3.9–9.0)	–2.1	0.033	0.29
Medical and social-status management	4.2 (2.9–8.7)	7.8 (6.7–9.0)	–3.1	0.002	0.42
Financial management	2.7 (1.0–6.7)	5.7 (3.1–9.0)	–1.9	0.046	0.27
Mobile phone use	7.8 (5.1–9.0)	8.7 (7.9–9.0)	–1.8	0.063	0.25
Social interaction	6.0 (4.1–7.4)	9.0 (7.1–9.0)	–4.2	< 0.001	0.57
Leisure	3.7 (2.5–6.7)	6.7 (5.6–9.0)	–3.1	0.002	0.42
Executive functions: cognitive scale	2.8 (2.1–3.5)	4.5 (3.9–4.9)	–4.1	< 0.001	0.56
Executive functions: behavioural scale	4.3 (3.5–4.6)	4.8 (4.7–5.0)	–3.9	< 0.001	0.54

TBI: traumatic brain injury; IQR: interquartile range.

**Table III:** Between-group comparisons of the Assessment of Participation and Executive Functions scores: second time-point

Assessment of Participation and Executive Functions	TBI group (*N* = 21) Median (IQR)	Stroke group (*N* = 24) Median (IQR)	*z*	*p* - value	Cohen’s *r*
Treatment-schedule management	9.0 (7.1–9.0)	9.0 (9.0–9.0)	–1.5	0.132	0.22
Medical and social-status management	9.0 (6.5–9.0)	9.0 (7.9–9.0)	–1.4	0.143	0.20
Financial management	9.0 (4.6–9.0)	9.0 (3.6–9.0)	–0.02	0.980	0.00
Mobile phone use	9.0 (7.9–9.0)	9.0 (8.7–9.0)	–0.9	0.354	0.13
Social interaction	8.5 (7.6–9.0)	9.0 (8.8–9.0)	–2.5	0.010	0.37
Leisure	6.7 (5.3–8.1)	7.5 (6.6–9.0)	–1.3	0.258	0.19
Executive functions: cognitive scale	3.7 (2.9–4.3)	4.7 (4.1–5.0)	–2.9	0.003	0.43
Executive functions: behavioural scale	4.6 (4.0–4.9)	4.9 (4.8–5.0)	–2.6	0.008	0.38

TBI: traumatic brain injury; IQR: interquartile range.

**Table IV:** Between-group comparisons of the other assessment scores: first time-point

Assessment	TBI group (*N* = 28) Median (IQR)	Stroke group (*N* = 26) Median (IQR)	*z*	*p*-value	Cohen’s *r*
Multiple Errands Test-Hospital version final score	11.0 (8.0–14.8)	12.0 (8.7–13.7)^a^	–0.3	0.762	0.04
Color Trails Test Part 1 *t* score	36.0 (20.0–47.0)	38.5 (25.5–46.5)	–0.4	0.631	0.05
Color Trails Test Part 2 *t* score	37.0 (20.0–50.7)	37.5 (26.5–48.0)	–0.2	0.827	0.02
Color Trails Test Interference Index	1.0 (0.5–1.3)	1.0 (0.7–1.4)	–0.3	0.729	0.04
Montreal Cognitive Assessment final score	23.0 (21.0–25.7)	25.0 (21.0–26.2)	–0.7	0.475	0.09

a One patient’s data are missing (declined to undergo the assessment).

TBI: traumatic brain injury; IQR: interquartile range.

**Table V:** Between-group comparisons in the other assessments scores: second time-point

Assessment	TBI group (*N* = 21) Median (IQR)	Stroke group (*N* = 24) Median (IQR)	*z*	*p*-value	Cohen’s *r*
Multiple Errands Test-Hospital version final score	6.0 (3.7–10.2)	8.5 (5.5–10.8)	–1.2	0.194	0.17
Color Trails Test Part 1 *t* score	35.0 (24.5–51.0)	43.5 (34.2–51.7)	–1.0	0.300	0.01
Color Trails Test, Part 2 *t* score	49.0 (34.0–54.5)	40.5 (27.2–53.0)	–1.2	0.219	0.17
Color Trails Test Interference Index	0.8 (0.4–1.2)	1.1 (0.8–1.5)	–1.8	0.062	0.26
Executive Functions Performance Test final score	5.0 (2.0–10.5)	5.0 (4.0–7.0)	–0.1	0.897	0.01

TBI: traumatic brain injury; IQR: interquartile range.

Table VI presents within-group differences between the first and second time-points in the A-PEX, MET-HV and CTT (Tables II–V detail the descriptive statistics). Significant differences were found in both groups between the time-points in all functional domains, the EF cognitive scale of the A-PEX and the MET-HV final score with a medium-to-large effect size. Significant differences were also found in the TBI group’s EF behavioural scale of the A-PEX and the CTT2 *t*-score, and in the stroke group’s CCT1 *t*-score.

**Table VI:** Within-group differences between first and second time-points in the assessments

Assessment	TBI group (*N* = 21)		Stroke group (*N* = 24)	
	*z*, *p*-value	Effect size	*z*, *p*-value	Effect size
Assessment of Participation and Executive Functions				
Treatment schedule management	–3.5, 0.000	0.76	–3.0, 0.002	0.61
Medical and social status management	–3.2, 0.001	0.69	–2.8, 0.005	0.57
Financial management	–3.4, 0.001	0.74	–2.6, 0.018	0.53
Mobile phone use	–3.1, 0.001	0.67	–2.5, 0.010	0.51
Social interaction	–3.7, 0.000	0.80	–3.2, 0.001	0.65
Leisure	–3.5, 0.000	0.76	–2.3, 0.019	0.47
Executive functions: cognitive scale	–3.4, 0.001	0.74	–2.3, 0.021	0.47
Executive functions: behavioural scale	–2.7, 0.006	0.58	–1.7, 0.077	0.34
Multiple Errands Test-Hospital version final score	–3.4, 0.001	0.74	–3.3, 0.001	0.67
Color Trails Test Part 1 *t* score	–1.1, 0.257	0.24	–2.8, 0.005	0.57
Color Trails Test Part 2 *t* score	–3.3, 0.001	0.74	–1.2, 0.217	0.24
Color Trails Test Interference Index	–1.3, 0.191	0.26	–0.7, 0.475	0.14

TBI: traumatic brain injury.

## DISCUSSION

The A-PEX was designed to evaluate executive functioning through leisure, social and IADL participation of adults hospitalized in inpatient rehabilitation facilities following brain injury. This study’s findings indicate that, unlike the MET-HV and CTT, the A-PEX can discriminate between TBI and stroke patients. According to the A-PEX, executive functioning in daily participation of patients post-TBI is worse compared with patients post-stroke, especially early in the rehabilitation process, which indicates unique executive functioning profiles that are manifested in the daily lives in the inpatient rehabilitation facility. In addition, the A-PEX provides information regarding changes in executive functioning as manifested in daily participation during inpatient hospitalization. The literature on assessment and treatment following brain injury lacks consistency in addressing ABI as a comprehensive condition or TBI and stroke separately (3, 5, 15–17). The current study indicates differences between the 2 types of injuries in how executive functioning are expressed in daily participation during inpatient participation. The A-PEX’s main functional domains distinguish the groups at the first time-point through activities such as knowing the results of medical examinations, initiating and organizing a friend’s visit and maintaining appropriate relationships with fellow patients. These complex, open-ended activities are cognitively demanding and require higher level executive functioning (2), both cold (e.g. monitoring, planning and cognitive flexibility) and hot (e.g. social judgment and emotional regulation). Considering the differences in executive functioning profiles and their prevalence rates between TBI and stroke, the wide spectrum of EF deficits in TBI compared with stroke may explain the differences in inpatient participation measured by the A-PEX. For instance, a patient’s ability to interact appropriately with others might be altered more by the low frustration rates, agitation and disinhibition that characterize TBI EF-deficits profiles than the hypoactivity or disinterest that characterizes stroke EF-deficits profiles (8,14).

At the first time-point, participants post-TBI performed worse on all the A-PEX domains except mobile phone use, particularly in medical and social status management and social interaction. Because mobile phone use is common and meaningful nowadays (40), especially among the young adults that comprised the TBI group, it may be that this functional domain is easier to master even in the presence of executive functioning deficits. This domain received the highest median score of all functional domains during the first assessment in both groups. The adults’ ability to use a mobile phone effectively at a very early phase in their rehabilitation process may be useful information in light of increasing research on using smartphones as assistive technology following brain injury (41).

At the second time-point, TBI group performance remained worse than stroke group performance in both EF scales and social interaction. This functional domain addresses the patient’s behaviour in the treatment room, such as engaging in an appropriate conversation with the therapist or other patients and asking pertinent questions regarding their rehabilitation process. This distinction between TBI and stroke is consistent with previous literature that found behavioural disorders more frequent post-TBI than post-stroke (2). The functional domain of social interaction encompasses social behaviour. Such behaviour involves several complex demands, such as interpreting and adapting to human and non-human sensory cues during interpersonal interactions, using complex communication pathways, interpreting information received by others and integrating past experiences. Several brain regions (cortical and subcortical) are activated together in this process. Some, such as the prefrontal cortex, hypothalamus and amygdala, have been identified; others have yet to be identified (42). Although we used reports of neural imaging from the medical records and did not perform them as part of the current study, it is plausible that the high percentage of patients with diffused injuries in the TBI group (78.6%) compared with the stroke group (26.9%) may have more affected brain regions and neural circuits involved in social behaviour, ultimately causing greater impairments in social interaction that persisted over time. The relations between the different injury mechanisms and social behaviour in actual daily participation should be further studied using imaging.

This study used an array of standardized EF assessment tools, all common in clinical and research use. In contrast to the A-PEX, these measures did not differentiate between TBI and stroke groups at either time-point, although both groups showed deficits according to these assessments. These results support the notion that assessing executive functioning in open-ended, actual, everyday functions may increase the sensitivity of the evaluation due to its ecological validity (21–23). These results indicate that the A-PEX may provide valuable information regarding executive functioning profiles, thus expanding theoretical and clinical knowledge regarding their manifestation in daily participation in TBI compared with stroke patients. A better understanding of executive functioning profiles during the subacute phase is essential, because their presence at this phase can significantly predict functional outcomes and the ability to reintegrate back into the community following TBI and stroke (43, 44).

While there are well-known assessment tools, such as the Assessment of Motor and Process Skills (AMPS; 45), the Performance Assessment of Self-care Skills (PASS; 46), the Perceive, Recall, Plan, Perform-System of Task Analysis (PRPP-assessment; 47) and the Canadian Occupational Performance Measure (COPM; 48) that are administered through a semi-structured interview and observation on performance of structured tasks, A-PEX administration involves a very different process. It capitalizes on the patient’s current setting (inpatient rehabilitation), a challenging environment requiring continuous planning, on-the-moment problem-solving, and decision-making to adapt to novel and changing environmental demands. All these require the use of a combination of executive functioning (6, 49). Furthermore, its administration and data collection are based on the clinician’s knowledge gathered over time, which provides information about a patient’s ability to perform open-ended activities in various occupations in a changing environment (50). The continuous naturalistic method of collecting inpatient participation data that entails activities that are changing constantly in dynamic environments, increases sensitivity to executive functioning profiles and changes over time and minimizes the learning effect.

Using the A-PEX may help the clinician to better understand the patient’s executive functioning profile and its influence on daily participation leading to an individually tailored treatment plan. The A-PEX structure, of EF scales and functional domains, provides the link between executive functioning and inpatient participation, which may guide the selection of the most appropriate metacognitive strategies based on which executive function most negatively affects daily participation. In addition, the activities that compose the A-PEX functional domains may be set as treatment goals, and since the A-PEX can be administered multiple times, it may also help the clinician adjust the treatment plan and goals according to the patient’s progress during the rehabilitation process. In terms of research, examining the efficacy of intervention approaches with the A-PEX, may provide accurate information regarding the influence of the intervention on the participants’ actual daily participation, which is considered the goal of rehabilitation.

Improved A-PEX and MET-HV scores from the first to the second time-points were observed in both groups. Seemingly, both the A-PEX and the MET-HV can inform the clinician about progress made during the inpatient rehabilitation in the subacute phase. However, a learning effect (51) might have influenced the results of the MET-HV. Because the MET-HV is a structured task that simulates real-world activities, it is difficult to determine whether the improvement stems from better EFs, a learning effect, or a mixture of both. The learning effect might be less prominent in the A-PEX, which is based on observations of multiple open-ended activities in a dynamic environment where the patients need to use their EFs to continuously adapt their performance (6). This is particularly true in an inpatient rehabilitation facility, which emphasizes independence and requires patients to participate actively in the rehabilitation process (49). Contributing factors to improved performance on the A-PEX could be spontaneous recovery or rehabilitation-induced improvement of executive functioning, as well as learning compensating strategies and implementing them in daily participation (52–55).

This study included a small sample size for 2 main reasons. First, due to the assessments administered along with the A-PEX, only patients able to undergo demanding EF assessments, especially during the early phases of rehabilitation post-ABI, were included in the study. Thus, low-functioning patients and patients with behavioural deficits who could otherwise be assessed using the A-PEX were excluded, which caused a recruitment bias. Future studies should consider broader inclusion criteria to capture the actual spectrum of ABI patients encountered in clinical conditions. This, together with imaging, may shed light on the characterization of different A-PEX profiles according to injury mechanisms, type and extant. Secondly, data for this study were collected during the COVID-19 pandemic, when lockdowns were implemented. At that time, admissions and hospitalizations in the inpatient rehabilitation facility were restricted, which limited eligible patients from participating in the study, also causing a recruitment bias. The COVID-19 restrictions and lockdowns also caused patients to drop out of the study due to early discharge from the rehabilitation facility. In addition, our division into the 2 injury types in this study was coarse; it did not address the different subtypes (i.e. dispersed, or localized injury) or regions of injury. Future studies should address the influence of injury characteristics, such as location and distribution on executive functioning profiles, and their manifestation in daily participation. In addition, data regarding upper extremity function using standardized tests was not collected, which may have limited our ability to evaluate its influence on the CTT performance. Confounding factors that may have contributed to the differences in profiles could be spontaneous recovery, differences in cognitive interventions received during rehabilitation, extant of caregiver support, level of self-awareness, and psychological factors, such as mood (52–55). It would be beneficial for future studies to examine these factors, and their possible effect on daily participation during inpatient rehabilitation.

In conclusion, this study provides initial information regarding the manifestation of deficits in executive functioning in activity performance and daily participation of TBI compared with stroke patients hospitalized in an inpatient rehabilitation facility. This information should be taken into account when conducting research. Moreover, these results indicate that assessing EFs through participation in daily leisure, social interaction and IADL activities may provide valuable information regarding the patient’s current state and their progress through rehabilitation. This information may support the construction of treatment plans to improve participation in daily life tailored specifically to the patient’s needs and abilities. Future studies should be conducted using a larger scale of data collection in order to determine the minimal significant clinical change on the A-PEX.
